# Tea-YOLOv8s: A Tea Bud Detection Model Based on Deep Learning and Computer Vision

**DOI:** 10.3390/s23146576

**Published:** 2023-07-21

**Authors:** Shuang Xie, Hongwei Sun

**Affiliations:** School of Automation, Hangzhou Dianzi University, Hangzhou 310083, China; 20063131@hdu.edu.cn

**Keywords:** tea bud, attention mechanism, YOLOv8s, deformable convolution, computer vision

## Abstract

Tea bud target detection is essential for mechanized selective harvesting. To address the challenges of low detection precision caused by the complex backgrounds of tea leaves, this paper introduces a novel model called Tea-YOLOv8s. First, multiple data augmentation techniques are employed to increase the amount of information in the images and improve their quality. Then, the Tea-YOLOv8s model combines deformable convolutions, attention mechanisms, and improved spatial pyramid pooling, thereby enhancing the model’s ability to learn complex object invariance, reducing interference from irrelevant factors, and enabling multi-feature fusion, resulting in improved detection precision. Finally, the improved YOLOv8 model is compared with other models to validate the effectiveness of the proposed improvements. The research results demonstrate that the Tea-YOLOv8s model achieves a mean average precision of 88.27% and an inference time of 37.1 ms, with an increase in the parameters and calculation amount by 15.4 M and 17.5 G, respectively. In conclusion, although the proposed approach increases the model’s parameters and calculation amount, it significantly improves various aspects compared to mainstream YOLO detection models and has the potential to be applied to tea buds picked by mechanization equipment.

## 1. Introduction

Tea trees are one of the most widely planted agricultural and forestry crops in the world. Currently, there are tea planting industries in more than 50 countries and regions around the world, and the planting scale is constantly expanding. Tea buds can be processed into tea and related products after maturation, which is one of the most consumed beverages in the world [1]. Tea picking is mainly divided into manual picking and mechanical picking. Manual harvesting is primarily based on the experience of workers, who rely on differentiating the color and shape of tea buds for selection. However, this approach suffers from low efficiency, consumes a significant amount of farming time, and is prone to overlooking buds during the harvesting process, which poses significant challenges to the labor force [2]. Mechanical picking solves the problem of slow picking speed and reduces labor costs, but the current detection still has relatively low precision, either in relation to recall or precision rates. Therefore, how to use computer vision technology to identify tender tea buds quickly and accurately will become a key issue for intelligent tea picking.

In recent years, with the advancement of science and technology, the emergence of image recognition and deep learning algorithms have had a significant impact on agriculture, including crop identification and yield prediction. Yang et al. [3] proposed a BCo-YOLOv5 network model for fruit object recognition and detection in orchards, introducing a bidirectional cross attention mechanism (BCAM) into the network, with a mean average precision $\left(mAP\right)$ of 97.70%, which improved the detection speed and precision of the model. Xie et al. [4] proposed an improved litchi detection model called YOLOv5-litchi for litchi detection in complex natural environments, which added several enhancements, including the convolutional block attention module (CBAM), small object detection layer, and weighted box fusion, enabling the model to locate and detect small targets accurately, as well as enhancing the detection performance for small objects. Compared to the original YOLOv5 network, the YOLOv5-litchi model achieved a remarkable enhancement in both the mean average precision $\left(mAP\right)$ and recall rate, with an improvement of 12.9% and 15%, respectively. Wu et al. [5] proposed a tea tree detection method based on the YOLOv7 network and multiple data augmentation techniques for detecting tea tree fruits in complex outdoor environments, which achieved a mean average precision $\left(mAP\right)$ of 96.03% and a precision of 94.76%, demonstrating excellent detection performance and strong generalization capabilities. Zhou et al. [6] proposed a deep learning fusion method based on visual perception and image processing for adaptive active localization, recognition, and determination of picking points for tea tree fruits, which successfully addressed the challenges of recognizing and localizing tea tree fruits in complex environments. Lai et al. [7] proposed an improved object detection model based on YOLOv7 and conducted a quantitative analysis of its maturity classification precision and detection performance in six different field scenarios. The average precision (AP) and recall rate achieved by the model was 95.82% and 89.83%, respectively, which was 2.71% and 3.41% higher than the original YOLOv7 model. The other relevant studies included the detection of tomato [8], wheat [9], pepper [10], kiwi [11], mango [12], and citrus [13], etc.

In the field of crop identification in agriculture, it is evident from the aforementioned studies that they achieved excellent detection performance because of the significant differences in color and shape between the recognized objects and background elements, such as leaves. However, there are complex environmental factors in tea gardens. For example, tea buds have a small volume, high density, and colors that are extremely similar to the surrounding environment. Therefore, accurately detecting tea buds in a complex environment is a challenging problem in this field. Guo et al. [14] proposed a segmentation method based on color and depth data from a stereo vision camera, which enabled more accurate detection of the shape of tea buds in both 2D and 3D spaces compared to 2D images. Additionally, they introduced a lightweight model called YOLOv4-lighted, which significantly reduced the inference time. The test results demonstrated an average positioning success rate of 87.10% and an average positioning time of 0.12 s. Gui et al. [15] proposed a lightweight tea bud detection network model based on YOLOv5, introducing the Ghost_Conv module and the bottleneck attention module (BAM). Compared to the original YOLOv5 model, the modified model achieved a 9.66% increase in average precision, along with a reduction of 52.402 G in floating-point operations and 22.71 M in the parameters. Cao et al. [16] proposed a tea bud detection algorithm that combined GhostNet and YOLOv5, which addressed the challenges of low detection precision and slow speed caused by the complex background and small target sizes of tea buds, achieving a detection precision of 76.31%. Yan et al. [17] proposed a new model called the Mask R-CNN Positioning of Picking Point for Tea Buds (MR3P-TS) that identified the contours of each tea tree and the position of the harvesting points. The model achieved a mean average precision of 0.449 and an F2 score of 0.313 for bud detection and a precision of 0.949 and a recall of 0.910 for the localization of the harvesting points. Li et al. [18] proposed a deep learning-based method for efficiently estimating tea yield by counting tea buds in the field using an enhanced YOLOv5 model with the squeeze and excitation network, which achieved a mean average precision of 91.88% for the test dataset, demonstrating its effectiveness and high precision in detecting tea buds. Cheng et al. [19] introduced an improved Mask R-CNN model for bud detection using a new anchor generation method and the CIoU loss function, which obtained 86.6% precision and 88.3% recall after transfer learning. Meng et al. [20] proposed an improved YOLOX-tiny model for tea bud detection by replacing its activation function with the Mish function and using a content-aware reassembly of the feature module to implement the up-sampling operation, with the mean average precision and recall rate for the improved model reaching 97.42% and 95.09%, respectively. Zhang et al. [21] replaced the feature extraction network for YOLOv4 and the standard convolution of the entire network with the lightweight neural network MobilenetV3 and the depth-wise separable convolution, and added a deformable convolutional layer and coordinate attention modules to the lightweight neural network, with the detection precision, recall, and AP of tea canopy shoots under different light conditions reaching 85.35%, 78.42%, and 82.12%, respectively. 

Summarizing the literature, there are still some issues in the current research. The existing target detection algorithms for tea buds simply increase the number for the dataset to improve the generalization performance of the model, but they do not take into account the quality of the image and the shape of the tea buds. During the process of detecting tea buds, the tea buds exhibit various shapes and have a high density with a small volume, and recognizing the different shapes of the same object requires the utilization of an extensive dataset to enhance the model’s learning capability on tea bud features. Additionally, some deep learning networks have high complexity and large model sizes, leading to a requirement for high computational resources. As the dataset size increases, the training and detection time for tea buds will be extended, which hinders the intelligent harvesting of tea buds. However, the Tea-YOLOv8s model proposed in this paper focuses on the informativeness of the tea bud images and the various growth postures of the tea buds, with higher precision. At the same time, it utilizes multiple image enhancements, supports multiple tasks and hardware platforms, and natively supports customized datasets.

In order to solve the above problems, this paper has three major contributions. First, because the growth of tea buds is affected by time and geographical location, the high-quality acquisition of datasets often consumes a lot of financial resources and manpower. Therefore, this paper uses brightness transformation, histogram equalization, gamma transformation, and homomorphic filtering to enhance the image, remove the noise and unnecessary details in the image, and highlight the features of tea buds more prominently, thus enriching the information content of the image and improving the detection precision. Second, because tea buds exhibit different growth postures, the shape and size of tea buds obtained in photographs are also different. Therefore, this paper introduces the variable convolution, attention mechanism, and improved spatial pyramid pooling, which learn the offset and weight of each sampling point, reduce the information dispersion, and expand the global interactive representation and the receptive field, so as to improve the spatial non-deformation and feature extraction ability of the model. Third, the Tea-YOLOv8s model is compared with other models in the YOLO series for tea bud detection and is subjected to ablation experiments, indicating that the Tea-YOLOv8s model has certain advantages.

## 2. Related Work

### 2.1. Attention Mechanism: Focusing on Selective Information

**Figure 1:**
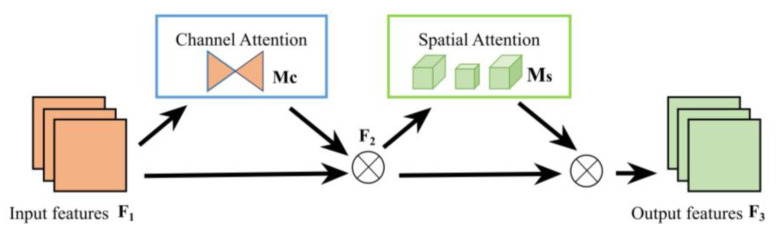
GAM attention mechanism module.

The attention mechanism has been proven to improve the precision of models [22]. In the process of exploring the application of the attention mechanism in computer vision, many excellent works have emerged, including SE [23], ECA [24], CBAM [25] and BAM [26]. GAM is a global attention mechanism proposed by Liu et al. [27] in 2021, which improves the performance of deep neural networks by reducing information diffusion and expanding global interactive representations. In addition to introducing the convolutional spatial attention submodule, GAM also introduces the 3D arranged channel attention with multilayer perceptron to retain information and amplify global cross-dimensional interactions, as shown in the Figure 1.

For the input feature map, $F_{1}$∈$R^{C\times H\times W}$ of the network, the formulas for calculating the intermediate state $F_{2}$ and output $F_{3}$ can be expressed as follows:

(1) $$F_{2}=M_{c}(F_{1})⊗F_{1}$$

(2) $$F_{3}=M_{s}(F_{2})⊗F_{2}$$

where $F_{1}$ represents the input feature map, $F_{2}$ represents the intermediate state, $M_{c}$ represents the channel attention module, $F_{3}$ represents the output feature map, $M_{s}$ represents the spatial attention module, and ⊗ represents the element-wise multiplication.

**Figure 2:**
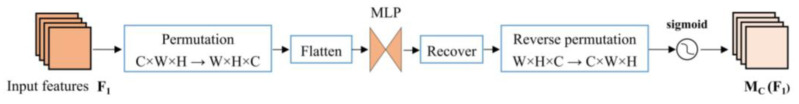
Structure diagram of the channel attention module.

In the channel attention module, GAM uses a three-dimensional arrangement to preserve information across all three dimensions. Specifically, the input feature F_1_ is first reshaped from C × W × H to W × H × C. Then, a two-layer fully connected layer (MLP) is used to amplify the interdependencies between the spatial and channel dimensions. Finally, the three-dimensional arrangement is reshaped back into the original C × W × H form, as shown in Figure 2.

**Figure 3:**
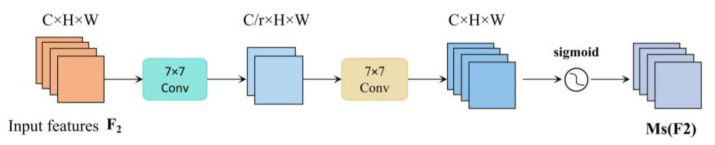
Structural diagram of the spatial attention module.

In the spatial attention module, GAM uses two convolutions to fuse spatial information in order to make the model focus on spatial information. At the same time, to avoid the loss of spatial information caused by pooling operations, pooling operations are not chosen, which further preserves the features, as shown in Figure 3.

### 2.2. Deformable Convolution

**Figure 4:**
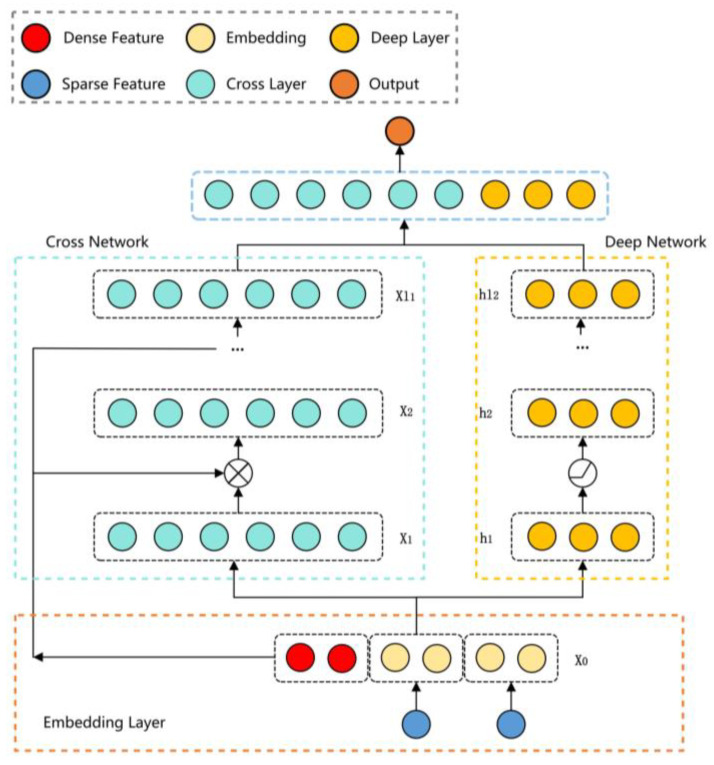
Visualization of DCNv2. ⨂ represents the cross operation, and 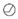 represents the activation function of ReLU.

In the scenes for object detection, an object to be detected may have various shapes. Therefore, recognizing the different shapes of the same object is a challenging task in object detection. The traditional strategies to solve this problem are usually divided into two categories: one is to enhance the model’s learning ability for such data by applying a large number of different data augmentations to the input image, and the other is to design some algorithms with invariance. Both of them are difficult to apply to complex environments like tea gardens. Therefore, in order to enhance the model’s ability to learn complex target invariances, the DCNv1 [28] deformable convolution was proposed, which can better adapt to the geometric transformation of the target through learning. However, the visualization results for DCNv1 show that the receptive field corresponding position exceeds the target range, resulting in the features not being affected by the image content. To address the aforementioned issues, DCNv2 [29,30] was introduced, as shown in Figure 4. By extending the deformable convolution, it learns the offsets and weights of each sampling point, thereby reducing the irrelevant interference information present in DCNv1 and improving the model’s ability to adapt to different geometric transformations. The formula can be expressed as follows:

(3) $$y\left(p\right)=\sum_{k=1}^{K}w_{k}\cdot x\left(p+p_{k}+{△p}_{k}\right)\cdot {△m}_{k}$$

where $w_{k}$ represents the weight of the *k*-th position, $p_{k}$ represents the pre-specified offset, ${△p}_{k}$ represents the offset of the deformable convolutional kernel, and ${△m}_{k}$ is a weight that can be used for end-to-end learning. Moreover, *x*(*p*) and *y*(*p*) represent the features at position *p* obtained from the input feature map *x* and the output feature map *y*, respectively.

### 2.3. Spatial Pyramid Pooling

**Figure 5:**
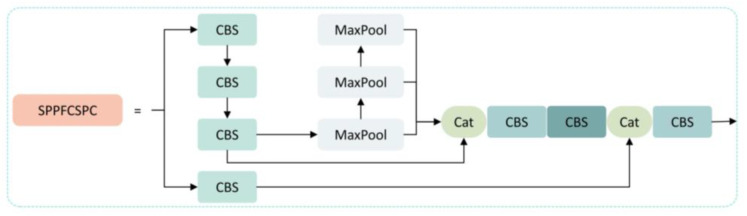
SPPFCSPC structure diagram.

In previous convolutional neural networks (CNNs), a fixed size image needed to be inputted for networks with predetermined structures, resulting in cropping, scaling, and other operations being performed when detecting images of various sizes, which decreased the precision of the detection. To address this problem, many excellent spatial pyramid pooling methods have been proposed, such as SPP [31], SPPF, ASPP, and SPPCSPC, which allow the network to input images of any size without the need for cropping, scaling, and other operations. This effectively avoids problems, such as image distortion caused by cropping and scaling operations on the image area, solves the problem of convolutional neural networks extracting repetitive features related to images, and improves the precision and speed of generating candidate boxes. SPPFCSPC [32] is an optimization of SPPCSPC based on the idea of SPPF, aimed at accelerating training and inference, as shown in Figure 5. By linking three separate pooling operations, less computation is required on the output results of the pooling layer with a smaller pooling kernel. This approach produces pooling layer results equivalent to those obtained with a larger pooling kernel, achieving a speedup while maintaining a constant perceptual field. The calculation formula for the pooling part can be expressed as follows:

(4) $$S_{1}\left(R\right)={MaxPool}_{k=5}^{p=2}\left(R\right)$$

(5) $$S_{2}(S_{1})={MaxPool}_{k=5}^{p=2}(S_{1})$$

(6) $$S_{3}(S_{2})={MaxPool}_{k=5}^{p=2}(S_{2})$$

(7) $$S_{4}=S_{1}⊛S_{2}\;⊛S_{3}$$

where *R* represents the input feature layer, $S_{1}$ represents the pooling layer result for the minimum pooling kernel, $S_{2}$ represents the pooling layer result for the medium pooling kernel, $S_{3}$ represents the pooling layer result for the maximum pooling kernel, $S_{4}$ represents the final output result, and ⊛ represents the tensor concatenation.

### 2.4. The Model Structure of the YOLOv8 Network

The YOLO series models are single-stage objective detection algorithms that use a single convolutional neural network (CNN) to simultaneously predict the class and location information of objects, requiring only one forward pass. Therefore, they have high precision and speed, making them particularly suitable for solving agricultural-related problems. With the development of agriculture, many excellent target detection algorithms have emerged, such as the Faster R-CNN model, the SSD model, and the Mask R-CNN model. In terms of speed, both the YOLO model and SSD model are single-stage objective detection algorithms, while the Faster R-CNN and Mask R-CNN models are two-stage algorithms, which results in the former group having a higher detection speed. In terms of precision, these four models have their own strengths and weaknesses depending on the specific application. The YOLO model is efficient in detecting small objects with real-time performance, Faster R-CNN is better suited for large image scenarios without real-time performance, the SSD model is more accurate in large object detection tasks, and Mask R-CNN performs better on complex and overlapping scenes. In terms of complexity, both the SSD and YOLO models have lower complexity and computational demand, making them easier to implement in parallel computation and hardware acceleration. Because of the need for multiple convolutions and forward and backward passes, the R-CNN and Mask R-CNN models have higher complexity.

**Figure 6:**
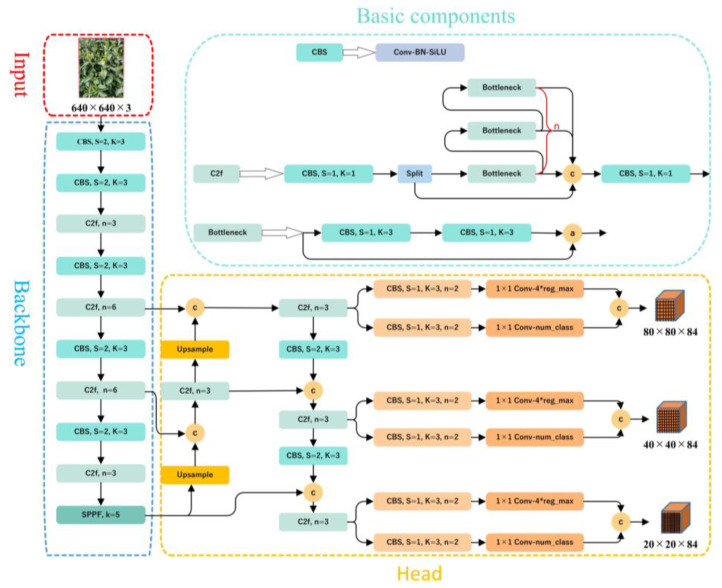
The architecture diagram for the YOLOv8s model. The * represents a multiplication sign, the letter c represents the addition of channel numbers, and the letter a represents the addition of feature maps.

YOLOv8 is the latest work in the YOLO (You Only Look Once) series, and it is also the most advanced object detection model. In 2015, YOLOv1 [33] was introduced, and the single-stage detection algorithm first appeared in people’s fields of vision. It effectively solved the shortcoming of the slow inference speed in the two-stage detection network and maintained a good effect on detection accuracy. YOLOv2 [34] further improved upon YOLOv1 by introducing batch normalization layers after each convolutional layer and removing the use of dropout. YOLOv3 [35] represented an advancement over the previous work, introducing significant improvements. Its key feature was the introduction of the residual module Darknet-53 and the Feature Pyramid Network (FPN) architecture, which allowed for the prediction of objects at three different scales and enabled the fusion of information across multiple scales. Since then, YOLOv4 [36], YOLOv5 [37], and YOLOv7 [38] have added many techniques based on version 3. Due to its leading performance, support for multiple tasks, well-developed toolchain for deployment, and flexible design, the latest YOLOv8 model, version YOLOv8s, is used in this paper, and the relevant code can be found on GitHub [39]. The YOLOv8s model consists of five parts: the input layer, backbone network, neck network, head network, and loss function, as shown in Figure 6.

**Figure 7:**
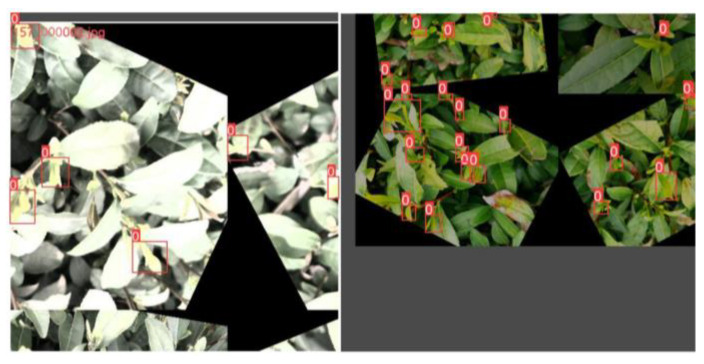
The effect of mosaic data augmentation. 0 and red boxes represent the category of tea buds.

The input layer of YOLOv8s uses three techniques: adaptive anchoring, adaptive image scaling, and mosaic data augmentation. During model training, YOLOv8s adaptively generates various prediction boxes based on the original anchor boxes and uses NMS to select the prediction boxes that are closest to the ground truth boxes. Due to the nonuniform size of the images, adaptive scaling resizes the images to a suitable standard size before inputting them into the network for detection, avoiding issues such as a mismatch between feature tensors and fully connected layers. Mosaic is a data augmentation method that concatenates four randomly scaled, cropped, and arranged images together to enrich the data and improve the network’s ability to detect small objects, as shown in Figure 7. In terms of improving small target detection, mosaic data augmentation can bring three benefits. First, small target detection typically requires a large amount of annotated data, but obtaining annotated data can be extremely expensive. By using mosaic data augmentation, multiple small images can be combined into a single large composite image, thereby increasing the size and diversity of the dataset without adding annotated data. Second, because small targets typically occupy only a small region of the image and are easily occluded by the background and other targets, small target detection is a challenging task. By using mosaic data augmentation, more variations and noise can be introduced during training, thereby enhancing the robustness of the model and its ability to handle different scenarios. Third, by using mosaic data augmentation, the model is exposed to a wider variety of scenarios and cases, which increases the generalization ability of the model.

The first convolutional layer of the backbone network is changed from a 6 × 6 convolution to a 3 × 3 convolution, and the idea of feature map splitting in CSPNet, and the residual structure are combined to propose the C2f module, which obtains richer gradient flow information while ensuring that it is lightweight. The YOLOv8 module still uses the SPPF module used in architectures such as YOLOv5, which serially passes the input through multiple maxpool layers, with a size of 5 × 5, on the SPP structure to avoid image distortion caused by cropping and scaling operations on image regions. Meanwhile, it solves the problem of convolutional neural networks extracting redundant features, greatly improves the speed of generating candidate boxes, and reduces computational costs.

The neck part of YOLOv8s still adopts the PAN-FPN structure to build the feature pyramid of YOLO, enabling sufficient fusion of multi-scale information. The convolution structure in the up-sampling stage of the PAN-FPN was removed, and the features output at different stages of the backbone were directly fed into the up-sampling operation. Additionally, the C3 module was replaced by the C2f module.

The head part of YOLOv8s has undergone significant changes compared to YOLOv5. It has been replaced with the currently mainstream decoupled head structure that separates the classification and detection heads and uses different branches for computation, which is beneficial for improving detection performance. Additionally, YOLOv8s has moved from anchor-based to anchor-free, avoiding the complex calculations and related hyperparameter settings of the anchor boxes, which has a significant impact on performance.

The loss function mainly consists of two parts: classification loss and regression loss. The classification loss is a varifocal loss (VFL), and the regression loss is in the form of a CIoU loss and distribution focal loss (DFL). Due to the use of DFL, the number of channels in the regression head has also become 4 × reg_max, while the number of channels in the classification head is the number of categories. The main improvement of the VFL [40] is the proposal on asymmetric weighting operations. For positive samples, *q* is used for weighting. If the gt_IoU of a positive sample is high, its contribution to the loss is larger, which allows the network to focus on high-quality samples. In other words, training high-quality positive examples has a greater impact on the AP than low-quality ones. For negative samples, weighting is used to reduce their contribution to the loss because the predicted *p* for negative samples is smaller. It becomes even smaller after reducing the power, which can reduce the overall contribution of the negative samples to the loss. The formula can be expressed as follows:

(8) $$\mathrm{VFL}(p,q)=\{\begin{array}{cc} -q(q\log(p)+(1-q)\log(1-p)) & q>0\\ -\alpha p^{\gamma }\log(1-p) & q=0 \end{array}$$

where *p* represents the predicted IoU-aware classification score (IACS), and *q* represents the target score.

DFL [41] stands for distance-based feature learning, which optimizes the probabilities of the two positions nearest to the target label *y* in a cross-entropy manner. It models the position of the box as a general distribution to enable the network to quickly focus on the distribution of positions close to the target location. The formula can be expressed as follows:

(9) $$\mathrm{DFL}(S_{i}+S_{i+1})=-((y_{i+1}-y)\log(S_{i})+(y-y_{i})\log(S_{i+1}))$$

(10) $$S_{i}=\frac{y_{i+1}-y}{y_{i+1}-y_{i}}$$

(11) $$S_{i+1}=\frac{y-y_{i}}{y_{i+1}-y_{i}}$$

where *y* represents the label, and $y_{i}$ and $y_{i+1}$ represent the nearest two to *y*($y_{i}$ ≤ *y* ≤ $y_{i+1}$).

The CIoU loss function [42] is based on the DIoU and adds the detection box scale, so the prediction box will better match the true box. The formula can be expressed as follows:

(12) $$CIoU=IoU-\frac{\rho^{2}(b,b^{gt})}{c^{2}}-\alpha v$$

(13) $$\alpha =\frac{v}{(1-IoU)+v}$$

(14) $$v=\frac{4}{\pi^{2}}{\left(arctan\frac{w^{gt}}{h^{gt}}-arctan\frac{w}{h}\right)}^{2}$$

where *ρ* represents the Euclidean distance between the centers of the prediction and truth boxes, *c* represents the diagonal distance between the minimum enclosing rectangle of the prediction and truth boxes, *b* and $b^{gt}$ represent the centers of the prediction and truth boxes, respectively, $w^{gt}$ and $h^{gt}$ represent the width and height of the ground truth box, respectively, and *w* and *h* represent the width and height of the prediction box, respectively.

Meanwhile, YOLOv8s abandons the previous IoU matching or one-sided ratio allocation method, and instead uses the Task-Aligned Assigner matching method to select positive samples based on the weighted scores for classification and regression. The formula can be expressed as follows:

(15) $$t=s^{\alpha }+u^{\beta }$$

where *α* and *β* represent the weight hyperparameters, *s* is the predicted score corresponding to the annotated category, *u* is the IoU between the prediction box and the truth box, and their product can measure the alignment degree.

## 3. Material and Methods

### 3.1. Image Acquisition

**Figure 8:**
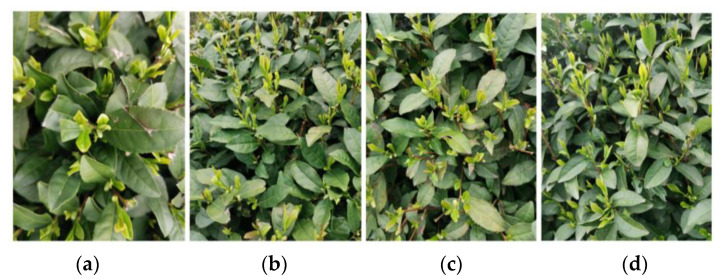
The images of the tea buds were acquired under different conditions: (**a**) near; (**b**) far; (**c**) top; and (**d**) side views.

This paper focuses on the image of tea buds in a complex environment, and the tea bud samples used in the study were collected from the Hangzhou Tea Research Institute in Zhejiang province, China, in 2022, using a high-definition camera on an OPPO Reno4 smartphone. In order to obtain tea bud images in a more realistic and high-quality way, the data collection was conducted outdoors in a sunny environment in mid-to-late March. During the process of collecting the image data of tea buds, we changed the camera’s angle and distance multiple times to capture the tea buds, and manually discarded some highly repetitive and blurry data. Finally, we obtained a dataset of 1238 original images, with approximately 14,856 tea buds in total. The collected tea bud images have various postures, including near, far, top, and side views. Some of the collected tea bud images are shown in Figure 8.

### 3.2. Data Pre-Processing

As image annotation is the most basic part of the process that can affect the model’s training and prediction, it is important to scientifically and reasonably annotate the tea bud images in the dataset. The annotation process for this experiment is divided into four steps. The first step is to manually remove some blurry original images. The second step is to upload the tea bud dataset manually to the EasyData platform (available online: https://ai.baidu.com/easydata, accessed on 12 February 2023) for intelligent annotation. The third step is to identify images that are not annotated or annotated incompletely by the EasyData platform and group them for manual annotation. The fourth step is to screen the annotated images, check for omissions and errors, and, finally, to save the labeled files in voc format.

**Figure 9:**
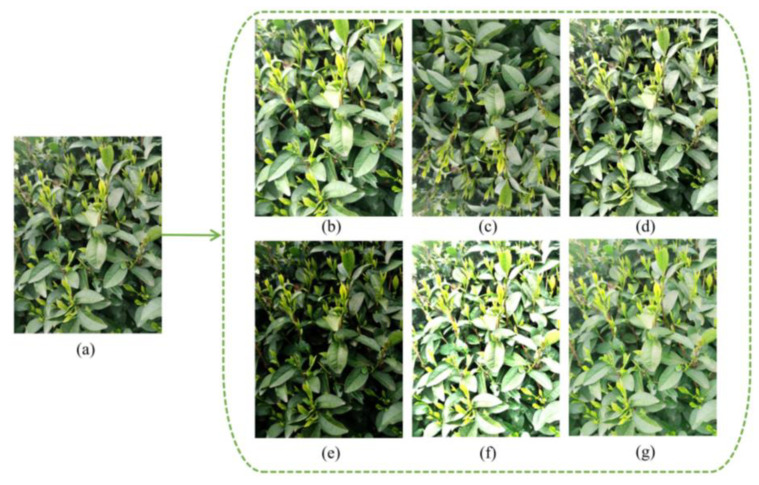
Results of the data augmentation: (**a**) original image; (**b**) brightness increased; (**c**) vertical mirror; (**d**) histogram equalization; (**e**) gamma transformation; (**f**) global homomorphic filtering; and (**g**) local homomorphic filtering.

In object detection networks with deep learning, the amount of data often affects the final recognition performance because too little data can lead to overfitting. For tea bud images, relying solely on self-collection is not enough, and the collection of tea bud images is also time limited, as it can only be done before and after the Tomb Sweeping Festival. In addition, field budding of tea bud images is also very time consuming. Therefore, data augmentation for tea bud images is necessary. The specific operation of data augmentation is to use relevant data augmentation methods to expand the existing tea bud images before making the dataset, thereby increasing the number of samples. This paper uses methods such as brightness transformation, flipping, histogram equalization, gamma transformation, and homomorphic filtering for data augmentation. Finally, the dataset is expanded to 4560 images, as shown in Figure 9. The image dataset is randomly divided into three groups, forming the model training, model validation, and testing datasets, with ratios of 70%, 20%, and 10%, respectively. These datasets will be used for model training and parameter optimization, and compared with the prediction results to evaluate the object detection performance of the model.

The idea of histogram equalization [43] is to transform the gray-level histogram of the original image from a relatively concentrated range of gray levels to a uniform distribution across the entire gray-level range. It performs a non-linear stretching of the image and reassigns the pixel values to achieve a roughly equal number of pixels within certain gray-level ranges. The formula can be expressed as follows:

(16) $$S_{k}=\sum_{j=0}^{k}\frac{n_{j}}{n}\;\;\;\;k=0,1,2,\cdot \cdot \cdot ,L-1$$

where ${\;S}_{k}$ represents the value after mapping the current gray level through the cumulative distribution function, *n* is the total number of pixels in the image, $n_{j}$ is the number of pixels in the current gray level, and *L* is the total number of gray levels in the image.

Gamma transformation is a non-linear transformation that expands the low and high gray scale range of an image, thereby correcting images that are too dark or too bright. By using different gamma values, it is possible to enhance the details of low or high gray scale parts. 

The formula can be expressed as follows:

(17) $$y=cx^{\gamma }$$

where *x* represents each pixel in the image, and *y* represents the output after linear transformation. Moreover, *c* represents the gray scaling coefficient, which is constant at 1.3 in this paper. In addition, *γ* represents the adjustment constant that controls the degree of scaling in the gamma transformation, which has a significant impact on the characteristics of the transformation function.

**Figure 10:**
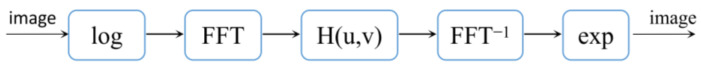
Flowchart of homomorphic filtering.

The specific implementation process for homomorphic filtering is to first take the logarithm of the image to be enhanced, and then perform Fourier transform. In the frequency domain, the homomorphic filter H$\left(u,v\right)$ is used for filtering, and finally the enhanced image is obtained through inverse Fourier transform and exponential transformation, as shown in Figure 10.

The ideal filtering effect in homomorphic filtering depends on the selection of the homomorphic filter function H$\left(u,v\right)$. H$\left(u,v\right)$ has two functions: one is to reduce the contribution of low-frequency (illumination) components, and the other is to increase the contribution of high-frequency (reflection) components. The final result is to compress the dynamic range of the image and enhance the contrast at the same time. In this paper, a Gaussian high-pass filter is used to construct the homomorphic filter function that can reduce the low-frequency components and increase the high-frequency components. The formula can be expressed as follows:

(18) $$H(u,v)=(\gamma_{H}-\gamma_{L})[1-e^{c(D^{2}(u,v)/D_{0}^{2})}]+\gamma_{L}$$

where $\gamma_{L}$ < 1 and $\gamma_{H}$ > 1, c represents a constant set to 1 in this paper, transitioning between $\gamma_{L}$ and $\gamma_{H}$, which is used to control the steepness of the slope in the filter function. $D_{0}$ is the cutoff frequency, and D$\left(u,v\right)$ is the distance from point $\left(u,v\right)$ to the center of the Fourier transform.

Previous studies [44] have shown that global homomorphic filtering can result in poor local contrast and over-enhancement of certain pixels. Therefore, to improve the local enhancement effect of the image, this paper adopts local homomorphic filtering [45], which involves dividing the image into a series of n × n sub-images, and then applying homomorphic filtering to each sub-image separately. Finally, the homomorphic-filtered sub-images are combined to form the enhanced image. Entropy is an important indicator for measuring the richness of image information. The larger the entropy, the more information the image contains, and the better the image quality. 

The local homomorphic filtering algorithm is employed to mitigate the impact of block artifacts on entropy values in this paper. To objectively evaluate the effectiveness of image enhancement, the local average entropy is utilized, which represents the average entropy value calculated for each individual sub-image. For a color image, the local average entropy is the average of the local average entropies for the R, G, and B components.

### 3.3. The Proposed Tea-YOLOv8s Model 

The Tea-YOLOv8s model combines multiple data augmentation methods and improved YOLOv8s. The main work is as follows: (1) image enhancement techniques such as mirroring, brightness transformation, histogram equalization, gamma transformation, and local homomorphic filtering are used to increase the amount of information, improve the image quality, and highlight the tea bud details; (2) the DCNv2 deformable convolution is added to the C2f module to enhance the model’s ability to learn the invariance in complex targets and reduce interference from irrelevant factors; (3) the global attention mechanism (GAM) is introduced to make the model focus on specific parts of the image, extract key information from the image, and ignore irrelevant information; (4) SPPF is replaced with SPPFCSPC spatial pyramid pooling, which increased the number of parameters, but the detection performance was better than SPPF.

**Figure 11:**
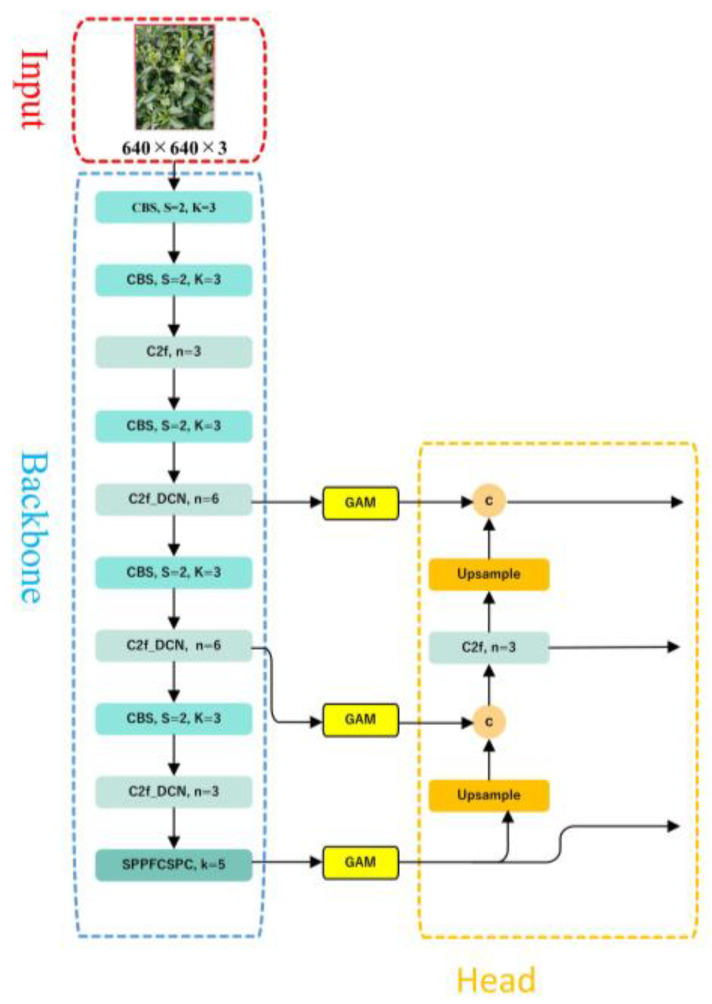
The improvements to the backbone network in YOLOv8s. The letter c represents the addition of channel numbers.

**Figure 12:**
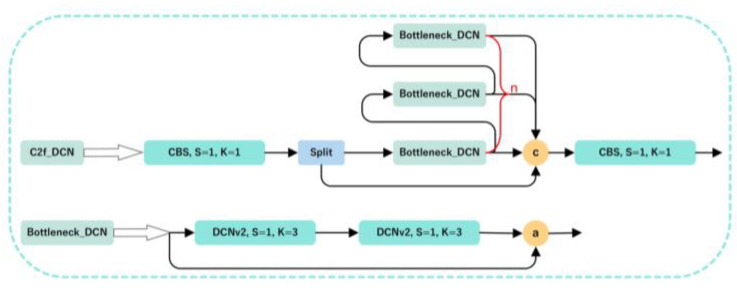
The structural diagram for the C2f_DCN module. The letter c represents the addition of channel numbers, and the letter a represents the addition of feature maps.

The backbone network is a key component for feature extraction, and this paper mainly proposes three improvements to the YOLOv8s backbone network, as shown in Figure 11. First, the C2f module in YOLOv8s is replaced with the C2f_DCN module, with the DCNv2 deformable convolution added to the C2f module. The C2f_DCN module still uses the CSP idea, two CBS modules, and n bottleneck modules. Different from the C2f module, this paper replaces the two CBS modules in the bottleneck module with DCNv2 deformable convolutions, as shown in Figure 12. By increasing the usage of deformable convolutions in the network and learning the weights of each sampling point, interference from irrelevant factors can be reduced. For certain undesired sampling points, their weights can be directly set as 0. Because the inference time will increase as the number of DCNv2 modules increases, this paper changes the last three C2f modules in the backbone network to C2f_DCN modules. Second, the GAM attention mechanism is added after the C2f_DCN module to further improve the feature extraction ability of the feature extraction network. As the attention mechanism is added to the backbone network, some of the original weights in the network will be destroyed, resulting in prediction errors. Therefore, this paper chooses to add the GAM attention mechanism to the feature enhancement part without destroying the original features of the network. Finally, this paper replaces the SPPF module with the SPPFCSPC module, which uses convolution and concatenation multiple times, on the basis of the SPPF module. Although this increases the number of parameters, it enlarges the receptive field, realizes multi-feature fusion, and improves the precision of the model.

### 3.4. Experimental Environment

All the experiments in this paper were conducted on the same computer. The hardware and software configurations for the computer and the specific information on the model training environment are shown in Table 1.

### 3.5. Training Parameters

The training parameters used during the experimental training process are shown in Table 2.

### 3.6. Evaluation Metrics

To evaluate the performance of the algorithm, this paper selects precision (P), recall (R), F1, and mean average precision $\left(mAP\right)$ as the evaluation metrics for the detection performance.

Precision represents the proportion of true positive samples among the samples that have positive prediction results. The formula can be expressed as follows:

(19) $$recision=\frac{TP}{TP+FP}$$

Recall represents the proportion of actual positive samples to all positive samples in the entire sample set that are correctly identified as positive by the model. The formula can be expressed as follows:

(20) $$Recall=\frac{TP}{TP+FN}$$

where *TP* represents the number of prediction boxes that match the tea bud picking points, *FP* represents the number of bounding boxes that did not predict the tea bud picking points, and *FN* represents the number of missed tea bud picking point bounding boxes. Therefore, it can be seen that precision represents the proportion of correct predictions among all the prediction results, while recall represents the proportion of correct predictions among all the true targets. The values for precision and recall are both between 0 and 1.

The *F*1 score represents the weighted average of the precision and recall. The formula can be expressed as follows:

(21) $$F1=\frac{2}{{Recall}^{-1}+{Precision}^{-1}}=2\;\cdot \;\frac{Precision\;\cdot \;Recall}{Precision+Recall}$$

Precision reflects the model’s ability to distinguish negative samples. The higher the precision, the better the model’s ability to distinguish negative samples. Recall reflects the model’s ability to recognize positive samples. The higher the recall, the better the model’s ability to recognize positive samples. The F1 score combines both the precision and recall. The higher the F1 score, the more robust the model.

The average precision (AP) represents the area under the precision–recall curve (PR curve), and the mean average precision $\left(mAP\right)$ represents the average of the APs for different categories. The formula can be expressed as follows:

(22) $$AP=\int_{0}^{1}P(R)dR$$

(23) $$mAP=\frac{\sum_{1}^{N}\int_{0}^{1}P(R)dR}{N}$$

where *N* represents the number of classes in the test set. Since there is only one class of tea buds in the dataset, AP *=* mAP.

## 4. Experiments and Results

### 4.1. Data Augmentation Performance

Due to the inclusion of a significant amount of background interference in the tea leaf dataset captured in natural scenes, as well as the influences from environmental factors, such as ground objects, lighting conditions, lens effects, and image quality degradation during transmission, the fine details of the tea leaves are not adequately highlighted in the image acquisition process, which severely affects the training of the model. Therefore, this paper adopts five data augmentation methods, including brightness transformation, mirroring, histogram equalization, gamma transformation, and homomorphic filtering, to denoise the collected dataset, enhance the relevant features, and suppress the irrelevant features in order to optimize the image quality. 

**Figure 13:**
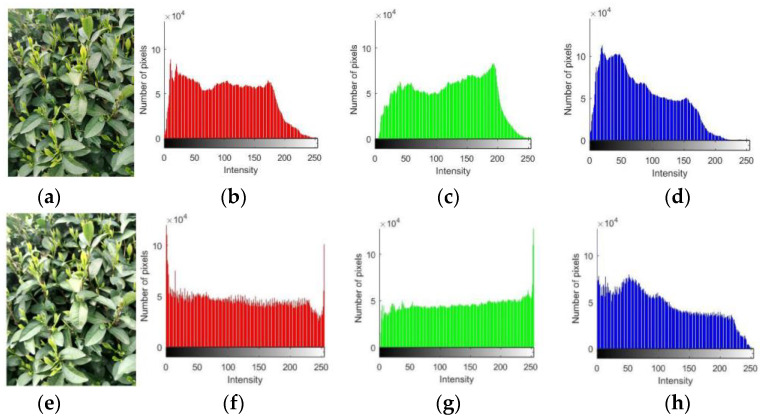
Results of histogram equalization: $\left(a,e\right)$ original image; $\left(b,f\right)$ the R component; $\left(c,g\right)$ the G component; and $\left(d,h\right)$ the B component.

From Figure 13, it is apparent that the details of the tea leaves are more prominently highlighted after histogram equalization. The brighter regions become brighter, and the darker regions become darker, resulting in an increase in the contrast and clarity in the image. Since histogram equalization deals with the intensity values for an image rather than its color components, this paper first converts the image color space from RGB to YCbCr, which separates the intensity value and color components, then performs histogram equalization on the intensity plane Y, and finally converts the generated YCbCr image back to RGB. By extracting the RGB values of the original image and the image after histogram equalization, it can be observed that the pixel values for the image are redistributed using the histogram equalization method, and the gray scale range is stretched. The number of pixels with the R component and the B component gray value of 0 is significantly increased, as well as the number of pixels with the R component and the G component gray value of 255. Additionally, the number of pixels in a certain gray scale range is roughly the same. Histogram equalization transforms the given image histogram distribution into a uniform distribution histogram, which is very useful for enhancing the details in the foreground and background that are either too bright or too dark.

**Figure 14:**
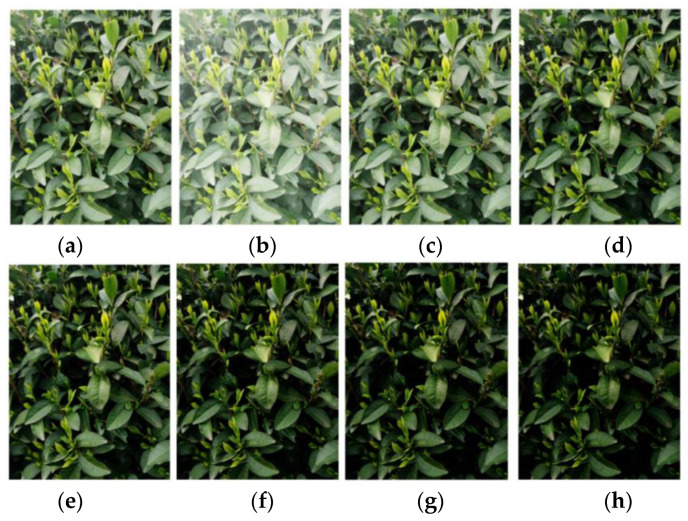
Results of the gamma transformation under different gamma values: (**a**) original image; (**b**) γ = 0.5; (**c**) γ = 1.0; (**d**) γ = 1.5; (**e**) γ = 2.0; (**f**) γ = 2.5; (**g**) γ = 3.0; and (**h**) γ = 3.5.

As shown in Figure 14, the images enhanced with different gamma values have some differences. When gamma is greater than 1, the higher gray level regions in the image are stretched, while the lower gray level parts are compressed. When gamma is less than 1, the gray level changes in the image are opposite to that when gamma is greater than 1. When gamma is equal to 1, the gray level transformation is linear.

**Figure 15:**
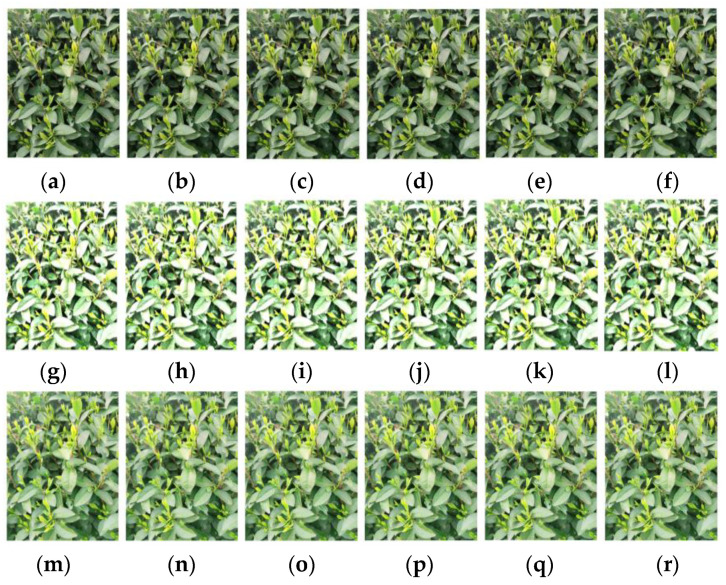
Results of homomorphic filtering and local homomorphic filtering under different block sizes: $\left(a,g,m\right)$ 8 × 8; $\left(b,h,n\right)$ 10 × 10; $\left(c,i,o\right)$ 12 × 12; $\left(d,j,p\right)$ 14 × 14; $\left(e,k,q\right)$ 16 × 16; and $\left(f,l,r\right)$ 18 × 18.

As shown in Figure 15, global homomorphic filtering causes over enhancement of the pixels in one part of the image, resulting in the loss of image details in another part. In contrast, local homomorphic filtering takes into account the local characteristics of the image. As a result of the enhancement, the image exhibits uniform illumination, moderate brightness and darkness, clear details, and a superior visual effect. As shown in Table 3, it can be observed that the original image undergoes a decrease in entropy after global homomorphic filtering, indicating a reduction in image information and quality. However, after applying local homomorphic filtering, the entropy increases, indicating an improvement in the image information and quality. Furthermore, it can be observed that the entropy also increases as the number of segmented sub-images increases, which indicates that a higher number of segmented sub-images leads to better image quality after local homomorphic filtering, with richer information and enhanced visibility of tea leaf details.

### 4.2. Comparison of the Overall Precision of the Network Models 

**Figure 16:**
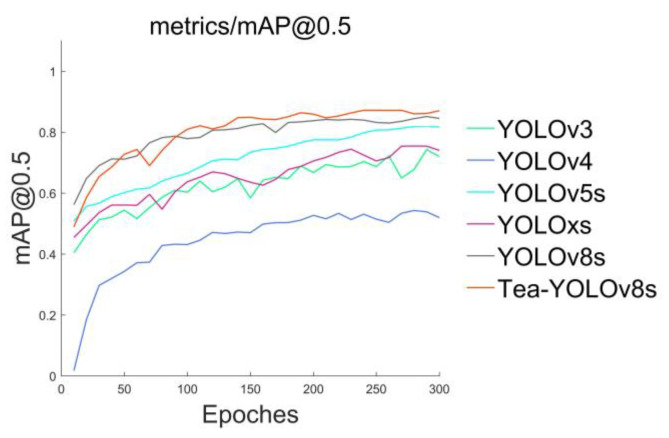
The ${mAP}_{@0.5}$ variation of the six object detectors.

**Figure 17:**
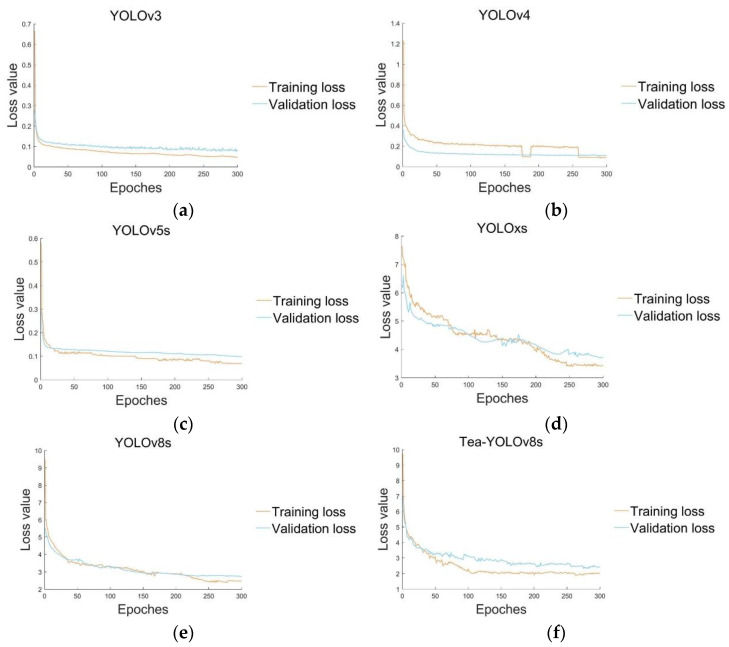
The loss variation curves for the six models: (**a**) YOLOv3; (**b**) YOLOv4; (**c**) YOLOv5s; (**d**) YOLOxs; (**e**) YOLOv8s; and (**f**) Tea-YOLOv8s.

We compared our improved model with five mainstream YOLO detection models and plotted the ${mAP}_{@0.5}$ curve for the different models, where ${mAP}_{@0.5}$ was recorded every 10 epochs, as shown in Figure 16. According to the six curves in different colors in the figure, it can be seen that the Tea-YOLOv8s model has certain advantages in the detection of tea buds. Other models, such as the YOLOv3, YOLOv4, YOLOv5s, YOLOxs, and YOLOv8s, have certain deficiencies in detection, as shown in Figure 17. Although YOLOv8s gradually approaches the Tea-YOLOv8s model in the mean average precision after 300 epochs, its convergence speed is slow and there are large fluctuations in early training. However, because the GAM attention mechanism has been added to enhance the feature distribution weight of the object to be detected in the spatial and channel dimensions and eliminate interference from useless features, the improved Tea-YOLOv8s model has a significantly accelerated convergence speed. Meanwhile, the ${mAP}_{@0.5}$ of the improved Tea-YOLOv8s model is better than that of the YOLOv3, YOLOv4, YOLOv5s, YOLOxs models, and the original YOLOv8s model.

In the process of detection, the annotated dataset for the tea buds is inputted into the YOLOv3, YOLOv4, YOLOv5s, YOLOxs, YOLOv8, and Tea-YOLOv8s detection models. Their precision, recall, F1 and ${mAP}_{@0.5}$ evaluation metrics are compared, as shown in Table 4. The Tea-YOLOv8s model proposed in this paper outperforms the other models in terms of precision, recall, F1, and ${mAP}_{@0.5}$. In terms of precision, the Tea-YOLOv8s model is 94.8%, which is 1.72%, 0.75%, 0.23%, 4.31%, and 4.76% higher than the YOLOv8s, YOLOxs, YOLOv5s, YOLOv4, and YOLOv3 models, respectively. In terms of recall, the Tea-YOLOv8s model is 81.23%, which is 5.1%, 26.62%, 9.21%, 66.05%, and 16.32% higher than the same models, respectively. In terms of F1, the Tea-YOLOv8s model is 87.49%, which is 3.73%, 18.39%, 5.72%, 61.49%, and 12.05% higher than the same models, respectively. In terms of ${mAP}_{@0.5}$, the Tea-YOLOv8s model is 88.27%, which is 3.59%, 14.34%, 6.40%, 36.31%, and 14.02% higher than the same models, respectively.

**Figure 18:**
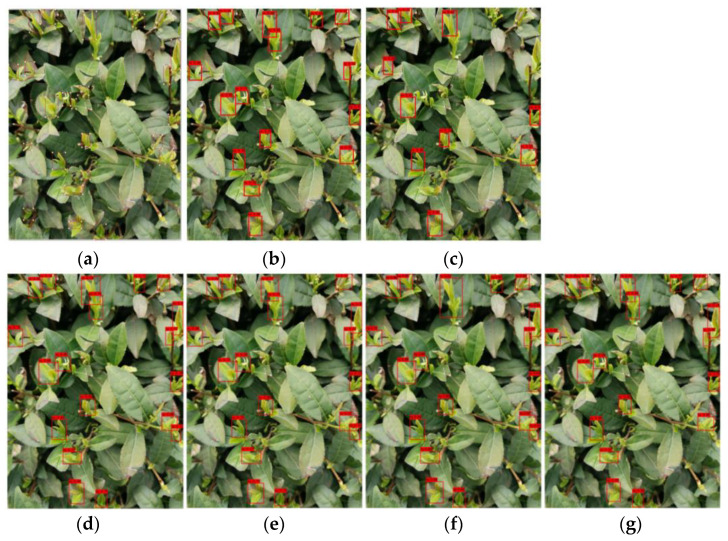
Test results for the six models: (**a**) label image; (**b**) YOLOv3; (**c**) YOLOv4; (**d**) YOLOv5s; (**e**) YOLOxs; (**f**) YOLOv8s; and (**g**) Tea-YOLOv8s.

In order to compare the tea bud prediction results for the various models more clearly and intuitively, this paper has compiled the prediction results for the various models, as shown in Figure 18. By adjusting the parameters, such as the learning rate, threshold, and epoch, continuously, the prediction results for each model are obtained. It can be seen that the detection results for these six models need to be improved, and missed and false detection phenomena exist in the actual detection. The Tea-YOLOv8s detection effect is the best, with the least number of missed and false detections and the highest precision among them. The other models have their own respective shortcomings. Compared with the label images, although the YOLOv3 model has a higher precision for some tea buds, the phenomenon of missed detection is higher, and the mean average precision is not as high as the Tea-YOLOv8s model. Among them, the YOLOv4 model has the worst detection performance, missing many tea buds and having low precision, which indicates that this model is not suitable for detecting small objects that are similar to the background. The performance of the YOLOv5s, YOLOxs, and YOLOv8 models is in the middle, and they can detect most of the tea buds with high precision. However, compared to the Tea-YOLOv8s model, the detection precision is still lower, indicating that the improved Tea-YOLOv8s model has a greatly improved detection performance. From the overall results for tea bud detection, it is difficult to detect obstructed, overlapping, and tender tea buds. At the same time, tea buds viewed from the side are better detected than those viewed from the top, indicating that attention should be paid to the season and angle when picking tea buds, which often leads to better detection and picking results.

### 4.3. Ablation Experiment

This section explores the impact of three improvement methods on the network model, and the data are shown in Table 5 and Table 6. We conducted eight experiments, each adding a different module, and compared them with the original YOLOv8s model based on indicators such as precision, recall, ${mAP}_{@0.5}$, ${mAP}_{@[0.5:0.95]}$, F1, layers, parameter, calculation amount, and inference time. The network with the GAM attention mechanism added is named YOLOv8s + GAM, the one with the DCNv2 deformable convolution added is named YOLOv8s + DCNv2, and the one with the SPPFCSPC spatial pyramid pooling added is named YOLOv8s + SPPFCSPC, and so on.

As the above data clearly shows, there is a significant difference between the ${mAP}_{@0.5}$ and ${mAP}_{@[0.5:0.95]}$ values, indicating that the YOLOv8s model has a relatively low degree of overlap between the prediction boxes and true boxes, and its detection precision needs to be improved. Compared with the original YOLOv8s model, the YOLOv8s + GAM model shows a decrease in precision by 1.1%. However, there is a significant improvement in the recall, ${mAP}_{0.5}$, ${mAP}_{@[0.5:0.95]}$ and F1 score. At the same time, the number of network layers, parameters, the calculation amount, and inference time also increase significantly. Although the precision of the YOLOv8s + SPPFCSPC and YOLOv8s + DCNv2 models decrease by 0.29% and 1.6%, respectively, and the improvement in the recall, ${mAP}_{@0.5}$, and F1 score are not as good as the model with the GAM attention mechanism added, the increasements in the number of network layers, parameters, the calculation amount, and inference time are minimal or even decreased, which is advantageous for hardware deployment. Compared to adding only the GAM module, the DCNv2 module, and SPPFCSPC module separately, the improvement effects of adding both the GAM and DCNv2 modules and the SPPFCSPC and DCNv2 modules are not very significant, but the calculation amount is reduced, indicating that adding the DCNv2 module can reduce the model’s calculation amount and improve its performance. The Tea-YOLOv8s model with all three modules added shows the highest precision, recall, ${mAP}_{@0.5}$, ${mAP}_{@[0.5:0.95]}$, and F1 score, which obtains the best detection performance. However, it also has the largest number of layers, parameters, calculation amount, and inference time. In summary, although our method increases the model’s parameters and calculation amount, it significantly improves its precision, which is a worthwhile improvement.

## 5. Discussion

Compared to directly using the YOLOv8s model for tea bud detection, the proposed Tea-YOLOv8s model in this paper significantly improves the precision of tea bud detection. However, there are still some tea buds that are not recognized, and the reasons can be summarized as follows. The first reason is related to the nature of the captured images. Factors such as sunlight [46] and wind can cause reflections and the overlapping of tea buds during the image capture process. Moreover, tea buds have a high density and small size. Tea buds that are too far away may result in blurry images, making it difficult to annotate and detect them. The second reason is related to the photographers themselves. The distance and angle between the camera and the tea buds are challenging to keep consistent. Different photographers may choose varying angles and distances for capturing the images, with side-angle tea buds often yielding better detection results [47]. The third reason is related to the Tea-YOLOv8s model itself, which has limitations in detecting small targets that have a high similarity to the surrounding environment.

Furthermore, the Tea-YOLOv8s model proposed in this paper demonstrates significant improvements in the precision, recall, ${mAP}_{@0.5}$, and F1 score compared to the original YOLOv8 model. However, this enhancement comes at the expense of increased parameters and the calculation amount. The traditional convolutions have limitations to the feature extraction and object localization because of their fixed scale and geometric structure [48]. By introducing the DCNv2 deformable convolution, these issues can be mitigated. However, the experimental results indicate that increasing the number of DCNv2 modules leads to a prolonged inference time, a considerable computational load, and an irregular memory access bottleneck, limiting its extensive deployment on edge devices [49]. Therefore, it becomes crucial to carefully replace the traditional convolutions with appropriate alternatives. Additionally, experiments have shown that introducing attention mechanisms [50], image enhancement methods [51], and enlarging the receptive field [52] can effectively improve detection performance. Nevertheless, these approaches come with the drawback of increased parameters and the calculation amount, making the deployment of the model challenging.

To apply this model to practical mechanized harvesting tasks involving tea buds, it can be deployed via a development board and integrated into a complete visual recognition system, with appropriate mechanical structures, designed for the tea harvesting machine. This implementation will enable the precise picking of tender tea leaves. Previous studies have developed computer vision systems using robotics and deep learning techniques for intelligent tea bud harvesting [53], a spatial positioning system for fruits [54], and the dimensions of tea bud harvesters [55], which provide theoretical support and insights on intelligent tea bud harvesting. However, there are still many aspects to consider in realizing a fully automated harvesting system. These include further reducing the calculation amount, parameters, and inference time of the Tea-YOLOv8s model, applying the model to different types of tea buds, and integrating it with hardware, which play a crucial role in the future automation of tea bud harvesting.

## 6. Conclusions

The tea garden presents a complex and diverse background, and manual tea leaf harvesting requires significant manpower and resources. Based on the fast detection capability of YOLOv8s, this paper proposes a Tea-YOLOv8s model primarily for the detection of tea buds. The model utilizes data augmentation techniques, such as mirroring, brightness variation, histogram equalization, gamma transformation, and local homomorphic filtering, to increase the amount of image information and improve the image quality, highlighting the details of tea leaves. The model has three improvements. First, DCNv2 deformable convolution is added to the C2f module, which utilizes more deformable convolutions and learns the offset and weight of the sampling points to enhance the model’s ability to adapt to geometric transformations and focus on the relevant image regions. Second, the GAM attention mechanism is added to the YOLOv8s backbone network to adjust the focus on different regions of the image and improve the model’s precision and detection efficiency. Finally, the SPPF module in the backbone network is changed to the SPPFCSPC module, which can increase the receptive field, achieve multi-feature fusion, and improve detection precision. The proposed Tea-YOLOv8s model in this paper is compared with five other object detectors using a test dataset and achieves favorable results across multiple metrics. The model achieves a mean average precision (${mAP}_{@0.5}$) of 88.27%, which is an improvement of 3.59%, 14.34%, 6.40%, 36.31%, and 14.02% over the popular YOLOv8s, YOLOxs, YOLOv5s, YOLOv4, and YOLOv3 models, respectively. However, compared to YOLOv8s, the inference time, the number of parameters, and the calculation amount increases by 18.1 ms, 15.4 M, and 17.5 G, respectively. In future work, we aim to further reduce the model’s parameters and the computational amount, while maintaining the same level of precision, design corresponding mechanical structures, and develop a complete visual recognition system to complement the model. Additionally, we plan to address the occlusion and overlapping of tea buds, incorporate temporal data from video sequences, and apply transfer learning techniques for model generalization.

## Figures and Tables

**Table 1:** Configuration and training environment.

Environmental Parameter	Value
CPU	AMD R9 4900 H
GPU	GeForce 3060 Ti
RAM	16 GB
Video memory	6 GB
Operating system	Windows10
Deep learning framework	Pytorch
Cudnn	Cudnn10.1
OpenCV	4.5.2

**Table 2:** Training parameters.

Parameter	Value	Parameter	Value
Learning Rate	0.01	Batch Size	4
Image Size	640 × 640	Epoch	300
Momentum	0.937	Mixup_prob	0.5
Optimizer	sgd	Weight Decay	0.0005

**Table 3:** Entropy values for homomorphic filtering under different block sizes.

Block Size	8 × 8	10 × 10	12 × 12	14 × 14	16 × 16	18 × 18
Original image	3.225	3.5879	3.8071	4.0067	4.1375	4.3077
Global homomorphic filtering	2.7675	3.0972	3.3048	3.4921	3.6157	3.7762
Local homomorphic filtering	3.4887	3.9484	4.1994	4.4251	4.5674	4.7571

**Table 4:** Model performance table under multiple indicators.

Models	P	R	F1	${mAP}_{@0.5}$
YOLOv3	90.04%	64.91%	75.44%	74.25%
YOLOv4	90.49%	15.18%	26.00%	51.96%
YOLOv5s	94.57%	72.02%	81.77%	81.87%
YOLOxs	94.05%	54.61%	69.10%	73.93%
YOLOv8s	93.08%	76.13%	83.76%	84.68%
Tea-YOLOv8s	94.80%	81.23%	87.49%	88.27%

**Table 5:** Ablation experiments with the modules.

Models	P	R	F1	${mAP}_{@0.5}$	${mAP}_{@[0.5:0.95]}$
YOLOv8s	93.08%	76.13%	83.76%	84.68%	56.93%
YOLOv8s + GAM	91.98%	79.96%	85.55%	86.25%	59.67%
YOLOv8s + SPPFCSPC	92.79%	78.94%	85.31%	86.12%	59.81%
YOLOv8s + DCNv2	91.48%	79.01%	84.79%	85.36%	60.27%
YOLOv8s + GAM + SPPFCSPC	93.77%	80.92%	86.87%	87.39%	64.30%
YOLOv8s + GAM + DCNv2	93.04%	81.17%	86.70%	86.84%	63.69%
YOLOv8s + SPPFCSPC + DCNv2	93.83%	79.38%	86.00%	86.19%	61.38%
Tea-YOLOv8s	94.80%	81.23%	87.49%	88.27%	65.72%

**Table 6:** Comparison of the prediction speed and computing resources.

Models	Layers	Parameters	Inference Time	GFLOPs
YOLOv8s	168	11.1 M	19.0 ms	28.4
YOLOv8s + GAM	201	19.7 M	27.5 ms	44.2
YOLOv8s + SPPFCSPC	178	17.6 M	21.6 ms	33.6
YOLOv8s + DCNv2	178	11.4 M	24.9 ms	25.0
YOLOv8s + GAM + SPPFCSPC	211	26.2 M	31.3 ms	49.3
YOLOv8s + GAM + DCNv2	211	20.0 M	35.7 ms	40.8
YOLOv8s + SPPFCSPC + DCNv2	188	17.9 M	28.2 ms	30.2
Tea-YOLOv8s	221	26.5 M	37.1 ms	45.9

## Data Availability

The data in this study are available upon request from the corresponding author.
